# The Myotendinous Junction—A Vulnerable Companion in Sports. A Narrative Review

**DOI:** 10.3389/fphys.2021.635561

**Published:** 2021-03-26

**Authors:** Jens Rithamer Jakobsen, Michael Rindom Krogsgaard

**Affiliations:** Section of Sports Traumatology, M51, A Part of IOC Research Center, Bispebjerg and Frederiksberg Hospital, Copenhagen University Hospital, Copenhagen, Denmark

**Keywords:** myotendinous junction, strain injury, Nordic Hamstring, Eccentric exercise, force transmission, injury prevention, hamstring strain injury

## Abstract

The incidence of strain injuries continues to be high in many popular sports, especially hamstring strain injuries in football, despite a documented important effect of eccentric exercise to prevent strains. Studies investigating the anatomical properties of these injuries in humans are sparse. The majority of strains are seen at the interface between muscle fibers and tendon: the myotendinous junction (MTJ). It has a unique morphology with a highly folded muscle membrane filled with invaginations of collagen fibrils from the tendon, establishing an increased area of force transmission between muscle and tendon. There is a very high rate of remodeling of the muscle cells approaching the MTJ, but little is known about how the tissue adapts to exercise and which structural changes heavy eccentric exercise may introduce. This review summarizes the current knowledge about the anatomy, composition and adaptability of the MTJ, and discusses reasons why strain injuries can be prevented by eccentric exercise.

## Introduction

The transition zone between skeletal muscle and tendon, the myotendinous junction (MTJ), has a key role in being the structure where muscle fibers interact with tendon. During muscle activity and especially during high effort exercise, large forces are transmitted from muscle fibers to the tendon through the MTJ (Huijing, 1999). The ability to transmit force is optimized by a unique architecture of the muscle fibers at their termination at the MTJ (Knudsen et al., 2015). Despite this, force applied to the MTJ can be so extensive, especially during eccentric loading, that strain injuries occur (Garrett, 1996). These are among the most frequent injuries in many popular sports (Orchard and Seward, 2002). There is intense research to develop models to predict who are at risk to be afflicted by these injuries, how to prevent them and on what the best rehabilitation strategy is, once they have occurred. Strangely, there has been a negligible focus on the ultrastructure of the human MTJ, which is where many injuries occur, and how the MTJ adapts to training and inactivity (Mjolsnes et al., 2004; Petersen et al., 2011; Seagrave et al., 2014; Van Der Horst et al., 2015). Even though such basic science studies would be highly relevant to understand the clinical aspects of strain injuries, the human studies have mainly explored the ultrastructure of the MTJ. The plasticity of the MTJ in relation to loading and unloading which is of particular interest for clinicians working with strain injuries, have been studied in animals exposed to running or weightlessness, and these activities are not easily transformable to human exercise situations.

The aim of this article is to review current knowledge about the ultrastructure and composition of the MTJ with focus on the human MTJ, strain injuries, with particular focus on hamstring strain injuries, and possible strategies for prevention. Also, the review suggests directions for research related to the MTJ in the future.

## Methodology

The literature for this review was found by searching PubMed. To identify studies regarding the structure of the MTJ and strain injuries, the following strategy was used:

(Myotendinous junction OR Muscle-tendon interface) AND (ultrastructure OR morphology OR force transmission OR strain injury OR hamstring strain injury).

To identify studies addressing strain injuries and prevention of hamstring strain injuries the following strategy was used: [(Muscle strain injury) OR (Hamstring strain injury)] AND (Injury prevention) NOT (sprain) NOT (tendinopathy) NOT (ACL).

All studies identified with the search strategies were manually evaluated by reading either the title, abstract or full article. In addition to articles identified from the search strategy, we used articles that we knew from our research on the MTJ. Studies regarding the neonatal or developing MTJ was not included in this review, and it has been reviewed thoroughly by others (Charvet et al., 2011, 2012; Valdivia et al., 2017).

The identified literature describing the basic structures of the MTJ and the adaptations at the MTJ following exercise seemed slightly dated. In contrast, there are many recent clinical investigations describing the preventive effects of various training protocols on the incidence of strain injuries, and the Nordic hamstring protocol is by far the most documented.

## Ultrastructure

**FIGURE 1:**
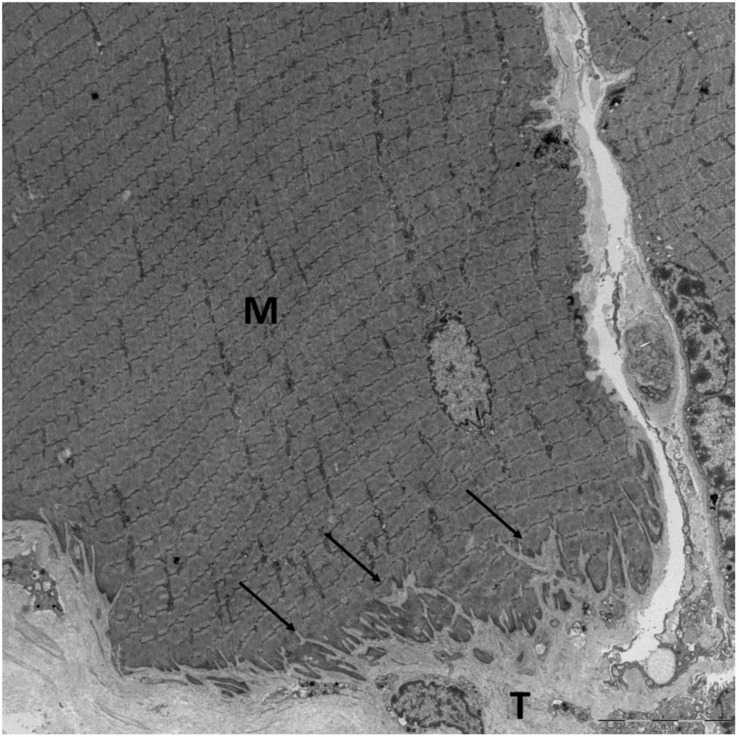
The MTJ of a human semitendinosus muscle fiber viewed with EM. The protrusions (arrows) from the tendon (T) into the muscle fiber (M) increase the contact area between the muscle and tendon. Scale bar is 10 μm. Image by Andreas B. Knudsen, made at the Centre for Integrated Microscopy (CFIM) at the Panum Institute in Copenhagen, Denmark, used by permission.

The ultrastructure of the MTJ in animals, show finger-like processes from tendon, protruding into muscle at the contact area between the two tissues (Trotter et al., 1985). By 3-D EM of the human MTJ these processes have been shown to be foldings of tendon, protruding into invaginations of the muscle membrane (Figure 1; Knudsen et al., 2015). This ultrastructure increases the contact area between muscle and tendon, making it possible for the contractile force from the myofibrils to be transmitted through a larger area. This results in a reduced amount of stress and higher breaking strength of the MTJ (Eisenberg and Milton, 1984; Tidball, 1991). In a model of frog semitendinosus MTJ it has been calculated that these protrusions increase the surface area by 13.2 times compared to a smooth cone-shaped junction (Tidball, 1984). Also, the folding structure increases the angle of force transmission, and consequently a high proportion of force is transmitted through shear stress (Tidball, 1983). For chicken latissimus dorsi muscles it has been reported that the surface area of the MTJ in type II muscle fibers is 40% larger compared to type I fibers (Trotter and Baca, 1987). Type II muscles are capable of producing higher forces than type I fibers, and it is good strategy that the transmission surface is larger in relation to type II fibers. However, whether this difference in surface area between muscle fiber types also exists in humans is not known. The correlation between muscle fiber type and risk of strain injury has been suggested previously (Garrett et al., 1984). In contrast to rats and mice, where many muscles are primarily composed of either type I or type II fibers, human muscles consist of varying proportions of both fiber types and in the most frequently injured muscles in humans, the hamstrings, rectus femoris and gastrocnemius, type II fibers are dominant (Polgar et al., 1973). Whether the surface area in the MTJ from these muscles is larger by development or can be increased through training, is unknown.

The plasticity (ability to adapt) of the MTJ to various loads has mainly been investigated in animals (summarized in Table 1) where most of the studies are slightly dated, and there is only one study investigating the human MTJ (Kannus et al., 1992; Tidball and Quan, 1992; Zamora et al., 1995; Roffino et al., 2006, 1998; Kojima et al., 2008; Palma et al., 2011; Curzi et al., 2012, 2013, 2016; Rissatto Sierra et al., 2018; Jacob et al., 2019; Pimentel et al., 2020). The protocols for the animal studies are very inhomogeneous, as the animals have been subjected to different regimens for loading and unloading, and as various species and muscles have been analyzed, which makes it impossible to draw one conclusion from these studies. In addition, the method to measure the area of the MTJ interface varies between studies (Table 1).

**TABLE 1:** Studies describing the effects of loading and unloading on the surface area of the MTJ with a description of species used, muscles and muscle fiber types and the method used for measurements.

**Studies describing the effect of increased load on the MTJ**					
**Author, year, species**	**Sample Size pr Group**	**Muscles/dominant fiber type**	**Intervention**	**Method of measurement**	**Results**

Kojima,2008,rat	5	Gastrocnemius/I Tibialis Ant./II	Running	Branching of fingers Angle of branching	↑
Curzi. 2012,rat	6	Gastrocnemius/I EDL/II	Running	Branching of fingers	↑
Curzi. 2016. rat	3	EDL/II	Running	Interface length/baseline(IL/B ratio)	↑
Sierra. 2018. rat	3	Soleus/I	Swimming	Length and thickness of invaginations and evaginations	↑
Jacob. 2019. rat	5	Sternomastoid/II	Swimming	IL/B	
Neto J, 2020 rat	10	Plantaris/II	Loaded ladder climb	Length and thickness of invaginations and evaginations	↑

**Studies describing the effect of unloading the MTJ**					

**Author, year, species**	**Sample Size pr Group**	**Muscles/dominant fiber type**	**Intervention**	**Method of measurement**	**Results**

Kannus, 1992. rat	8	Gastrocnemius/II Soleus/l	Immobilization 1 and 3 weeks	Length of surface area in relation to muscle fiber diameter	↓
Tidball 1992. rat	6	Plantaris/lI	Space flight -4 davs	Folding factor	↓
Zamora. 1995. rat	2 5	Soleus/I Plantaris/lI	Limb suspension for 8, 18 and 29 davs Space flight 14 days	No quantification	Only minor changes observed in Plantar is MTJ’s. thinning and lengthening of the foldings at MTJ of Soleus MTJ s
Roffino, 1998. rat	8	Soleus/I	Space flight 14 days	IL B ratio-named normalized interface values by the authors.	↑
Roffino, 2006.monkey	2	Soleus/I +II	Space flight 14 days	No qualification	-
de Palma, 2011,human	12 Unloaded 3 controls	Gastrocnemius/I+II	Bed rest (mean 60 months) in chronic diseased patients	Length of surface area n relation to muscle fiber diameter	↓
Curzi,2013,rat	4	Plantaris/II	Unloading	IL/B ratio Length of primary digits Brancing of fingers	↓

**In most studies the assessments have not been performed blinded to study group. Most studies provide information about the number of foldings/invaginations measured per group and not per animal and have not provided information about the number of slides from each animal. Because of this, the results should be interpreted with some caution. Arrows indicate whether an increase (↑) or a decrease (↓) in the measures was reported. EDL, Extensor Digitorum Longus.**

Overall, in the rat MTJ there is evidence of an increasing complexity of the foldings as response to a period of intensified loading (by running), with higher number of branches and longer foldings (Kojima et al., 2008; Curzi et al., 2013, 2016). This indicates that the MTJ adapts to load by increasing the junctional interface. Higher intensity of loading provided as fast running has shown significantly greater increases in the surface area of the MTJ compared to a lighter intensity running intervention (Curzi et al., 2016). This positive effect of load was not seen on the length and thickness of the evaginations when compared in a model of ladder climbing and loaded ladder climbing (Pimentel et al., 2020). It would be relevant in future studies to implement various training variables, such as intensity, and study the effect on the surface area of the MTJ.

There are varying results on the effect of unloading (Table 1). The rat MTJ shows a smaller interface between muscle and tendon in groups after 3 weeks of unloading compared to control groups, and this is effected by shortening of the foldings. The surface area of the MTJ is almost 50% in the unloading groups, independent of muscle fiber type (Kannus et al., 1992), and similar changes have been recorded after only 4 days of weightlessness during a spaceflight (Tidball and Quan, 1992). Contrary, the length of foldings in MTJs from rats that had been exposed to a 14 days spaceflight and weightlessness was 58% higher than in controls (Roffino et al., 1998).

These conflicting results may be explained by the method to calculate the surface area, namely as the ratio between the length of foldings at the MTJ and muscle fiber diameter. It is reasonable to assume that 14 days of weightlessness results in a higher degree of muscle fiber atrophy than 4 days, and if the length of the foldings remain constant, the calculated surface area increases with increasing length of loading. This may explain the surprising higher surface area of MTJ in rats after 14 days space flight.

The effect of unloading on the surface area of the MTJ in humans has been studied in 12 elderly patients who had a femoral amputation after being bed based for a long time period due to chronic disease. When compared to specimens from legs of 3 young persons who had acute amputations after accidents, there was a reduction in the surface area of the MTJs in the bed based patients (Palma et al., 2011). However, it is not known to which degree this was a result of unloading, the age difference or the chronic disease conditions.

Reduction of the surface area between muscle and tendon as a consequence of unloading can theoretically weaken the MTJ by making it less capable to transmit load – and thereby more susceptible to injuries. This may be the reason why elite soccer players, who had less than 10 training sessions before they participated in a competitive match after an injury period, had an increased risk of a subsequent injury (Bengtsson et al., 2019). For every additional training session before returning to match, the odds for an injury were 7% lower. This indicates that after a period of reduced activity, loading of the hamstrings MTJ should be implemented in training as soon as possible before full activity is resumed.

## Composition of the MTJ

Through invagination of the muscle fiber membrane the cytoskeleton from the muscle fibers is in contact with the extracellular matrix (ECM) which connects muscle to tendon: the cytoskeletal connection—from actin to the ECM (Henderson et al., 2017). In brief, intramuscular bundles of actin filaments from the last A-band attaches at the cytoplasmic side of the sarcolemma to either dystrophin, which connects with the transmembrane dystroglycan complex, or the vinculin-talin-integrin complex (Shear and Bloch, 1985). These complexes penetrate the sarcolemma and attach actin on the intracellular side with laminin and collagen fibrils on the other, providing the path for force transmission from muscle fiber to tendon. Dystrophin and integrins therefore represent the physical structure through which force is transmitted. Knockout studies on mice have shown that absence of either dystrophin or integrin severely reduces the strength of muscle and increases its susceptibility to injuries (Guo et al., 2006). Interestingly, it has been observed, that by increasing the levels of integrin, the negative effects from the knockout of dystrophin are largely compensated (Hakim et al., 2013). This finding is clinically relevant for patients with Duchennes muscular dystrophy, since absence of dystrophin is the main pathogenesis of the disease. However, in addition to impairing the force transmission through the MTJ, called the linear force transmission pathway as it occurs in line with the sarcomeres, absence of dystrophin also affects lateral force transmission (Ramaswamy et al., 2011). Through this pathway force is transmitted from the contractile elements in the muscle fiber laterally through costameres to the endomysium (Pardo et al., 1983; Ervasti, 2003). There are junctions between the endomysium and perimysium, suggesting that lateral force transmission might occur through these junctions to the perimysium, which continues as endotenon in the tendon (Passerieux et al., 2006, 2007). It is not known if the lateral force transmission is changed by loading and unloading. However, an increase in lateral instead of linear force transmission through the MTJ would relieve the MTJ of some of the extra stress related to increasing training load.

**FIGURE 2:**
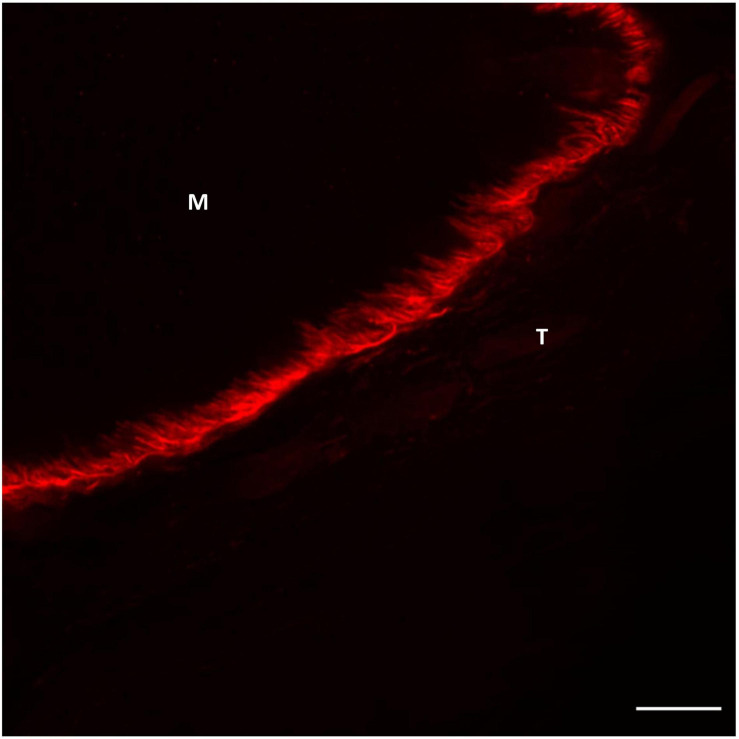
Confocal image of human semitendinosus MTJ showing the highly folded muscle membrane at the interface between muscle (M) and tendon (T). Collagen XXII labeling (red) the foldings connecting the tendon (T) with the muscle (M) The nuclei are stained blue (DAPI). Scale bar is 10 μm. For staining protocol and details regarding image acquisition (see Supplementary File).

**FIGURE 3:**
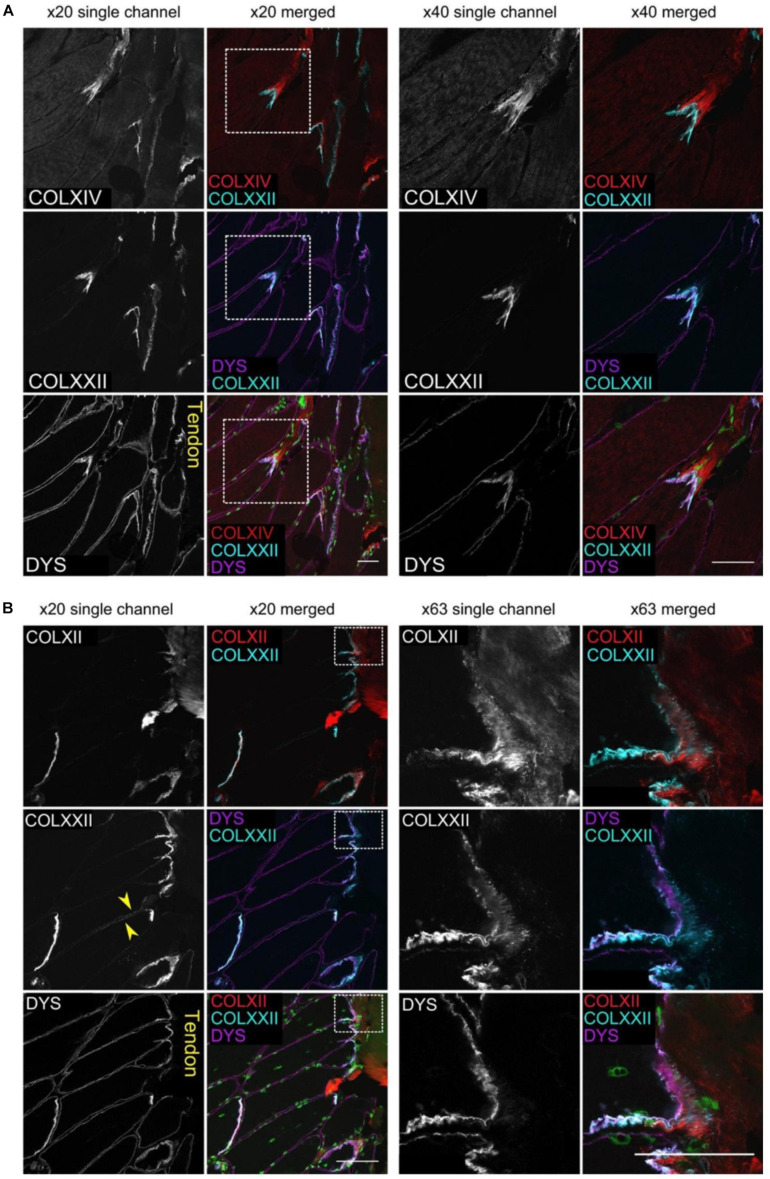
The figure shows multiple images of human gracilis MTJ stained with immunofluorescent antibodies against collagen XXII, dystrophin and either collagen XIV **(A)** or XII **(B)**. The muscle fibers show to the left and the tendon to the right. Dystrophin is located closest to the muscle fiber with collagen XXII immediately outside the muscle membrane. Collagen XIV and XII are present next to collagen XXII on the outside of the muscle fiber. Scale bars are 50 μm. The figure is from Jakobsen et al. (2017), used by permission.

It has been demonstrated by confocal imaging of MTJ that collagen type XXII is exclusively present in the foldings immediately on the tendon side of the muscle fiber membrane (Figures 2, 3; Jakobsen et al., 2017). The specific function of this collagen is not known, but knockout studies in zebrafish have shown a reduced potential for force transmission as well as an increased risk of ruptures at the MTJ when collagen XXII is absent (Charvet et al., 2013). Collagen XXII is located at tissue junctions and is not found elsewhere in skeletal muscle (Koch et al., 2004). Therefore, it is a marker to identify the MTJ, which is helpful, for instance in wide field imaging. Next to collagen XXII on the tendon side of the MTJ is a variety of collagens, including the smaller FACIT (Fibril Associated Collagen with Interrupted Triple Helices) collagens XII and XIV (Figure 3; Jakobsen et al., 2017). Future studies must determine the functions of these various collagens.

## Effects of Exercise on the Composition of the MTJ

There is incomplete knowledge about the effects of exercise on the composition and ultrastructure of the MTJ.

In human muscle the concentration of integrin type α7β1 increases following eccentric exercise (De Lisio et al., 2015). This integrin is also located at the MTJ, and because integrins are responsible for direct force transmission from muscle to tendon, increasing levels may be a way to prepare MTJ for increasing stress (Boppart et al., 2006). Similarly, in mice a single session of multiple eccentric contractions induces a significant increase of vinculin and talin in the soleus and plantaris muscles. Similar results are found in rats exposed to running with a higher expression of the mRNA for vinculin (Curzi et al., 2016). Although these proteins are found in the MTJ, they are not exclusively seen in this region, indicating that the increase in expression following exercise could arise from different regions of the muscle. Like the MTJ, both the neuromuscular junction and the costameres involved in lateral force transmission contain vinculin (Pardo et al., 1983; Ervasti, 2003). Both studies analyzed whole muscles and parts of tendon meaning that the increases in vinculin and talin are not necessarily seen at the MTJ. However, these two proteins are known to be present to a high extent at the MTJ but also neuromuscular junction, meaning that the increase following exercise is likely to arise from the MTJ where they are both elements in the chain of proteins that are responsible for force transmission through the junction (Frenette and Côté, 2000). Increase in expression of vinculin was also observed in rats exposed to a running intervention. However, it is not known if the increased levels of these proteins after exercise result in strengthening of the MTJ. It could be expected that the amount of larger collagens in the human MTJ would increase following heavy resistance exercise, but this has not been demonstrated for collagen I, III and VI (Jakobsen et al., 2018). However, the FACIT collagen type XIV increases in the endomysium surrounding the muscle fibers closest to the MTJ following exercise (Jakobsen et al., 2017). Like the MTJ, the endomysium contains a wide variety of collagens, and collagen XIV might be responsible for anchoring the larger collagen fibrils to form stronger networks.

The effects of various exercises on the length of the distal sarcomere in rat MTJs have been studied. Whether these lengths are important for the MTJ and risk of injury is unknown. Theoretically shorter sarcomere length, not to be confused with fascicle length, would be beneficial at the MTJ since they are more resistant to stretch during heavy loading, due to more overlapping cross-bridges between actin and myosin (Morgan et al., 1991; Rassier et al., 2020). In rats exposed to swimming exercise the distal sarcomeres of the sternomastoid MTJ are significantly shorter than in resting control rats (Jacob et al., 2019). However, the opposite is found following the same exercise intervention in rats of similar age, in the soleus muscle (Rissatto Sierra et al., 2018). So, there is conflicting evidence.

Following loaded and unloaded ladder exercise the distal sarcomere of rat soleus muscle is found to be shorter than in control rats. In contrast, the distal sarcomere from the plantaris muscle from the same animal is longer compared to control rats (Rocha et al., 2020). Whether the inhomogeneous effects of exercise on the length of the distal sarcomere at the MTJ is due to differences in the exercise regimens used, or that different muscles are loaded differently during the exercises, is not known. Future research is needed, preferably with the use of eccentric exercise as the chosen exercise modality since this is what has been shown to have a positive effect on the risk of strain injury (as discussed in detail later). Results from each animal or person should be pooled before statistical analysis of group values (instead of pooling all results in the groups). Finally, the person analyzing data should be blinded to the intervention. In a number of earlier studies (Kannus et al., 1992; Tidball and Quan, 1992; Zamora et al., 1995; Kojima et al., 2008; Curzi et al., 2012, 2013, 2016; Rissatto Sierra et al., 2018; Jacob et al., 2019; Pimentel et al., 2020) there is no certainty that data have been acquired after such basic principles.

## Cell Turnover

**FIGURE 4:**
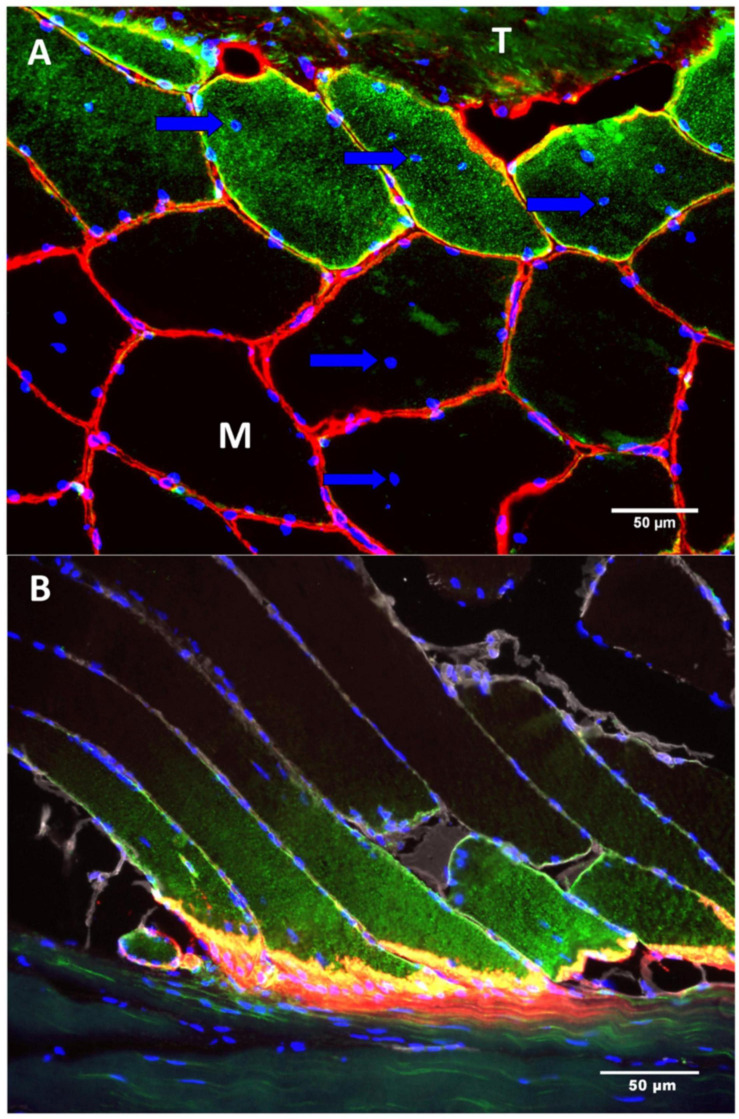
The figure shows two sections **(A,B)** of a human semitendinosus muscle stained for NCAM (green), collagen IV (red) and DAPI (Blue—indicating the nuclei). **(A)** In the top of the image there is tendon (T) and below is the muscle (M). The muscle fibers located closest to the tendon express signs of remodeling by containing centralized myonuclei (arrow) as well as NCAM(CD5S6) staining of the cytoplasm. **(B)** As the muscle fibers approach the MTJ the immunoreactivity of NCAM increases whereas no staining is seen in the fibers in the upper part of the image, farthest from the MTJ. Scale bar is 50 μm. Images are reprinted with permission from Jakobsen et al. (2018).

The MTJ is not a resting structure. There is a very high rate of remodeling in the muscle fibers near the MTJ (Jakobsen et al., 2018). Approximately half of the fibers adjacent to the MTJ contain one or more centralized nuclei and stain positive for the Neural Cell Adhesion Molecule (NCAM), indicating remodeling or formation of new muscle fibers (Figure 4A; Jamali et al., 2000; Chargé and Rudnicki, 2004). Contrary, in the muscle belly only a small percentage of the muscle fibers contain centralized nuclei (Dreyer et al., 2006). Even in muscles that have not exercised, almost 50% of the fibers at the MTJ contain centralized nuclei, indicating extensive remodeling in this area. On cross-section images the increased proportion of fibers with centralized nuclei normalizes just few fibers from the MTJ. Similarly, longitudinal sections have shown that the same muscle fiber has a higher expression of NCAM at its distal end near the MTJ compared to further from the MTJ, suggesting regional differences in protein expression across the same muscle fiber (Figure 4B). This remodeling involves different cell types, including satellite cells, fibroblasts and macrophages (Murphy et al., 2011; Bentzinger et al., 2013).

Satellite cells surround the periphery of resting muscle fibers, ready to be activated. Upon activation, they proliferate into daughter cells that can be donated as new myonuclei or fuse to form new myotubes. In skeletal muscles, satellite cells proliferate when muscles are exposed to resistance exercise (Kadi and Thornell, 2000), and even a single exercise session is enough to induce this (Bellamy et al., 2014). However, the high rate of remodeling at the MTJ is not associated with a concomitant increase in concentrations of satellite cells. In fact, the number of satellite cells near MTJ is the same as in muscle tissue far from the MTJ, and the concentration of satellite cells is unaffected by 4 weeks resistance exercise (Jakobsen et al., 2018). This indicates that satellite cells at the MTJ respond differently than those in muscle. However, it could be a dynamic phenomenon: the extremely high rate of remodeling at the MTJ could initiate a high demand for satellite cells, and the resulting, newly formed daughter cells could fuse immediately with a muscle fiber. If this is the case, the increase in the satellite cell pool will not show in a static cell count. However, additional studies are needed to fully understand the dynamics of satellite cells at the MTJ. While satellite cells have been shown to be detrimental for optimal muscle regeneration and repair they are not the only cell type involved (Joe et al., 2010; Sambasivan et al., 2011; Wang and Rudnicki, 2012). Inflammatory cells, such as macrophages and neutrophils, participate in this complex process, as well as fibroblasts (Toumi et al., 2006; Tidball and Villalta, 2010). Interestingly, fibro-adipogenic cells (FAPs) which are the precursor cells for fibroblasts and adipocytes, have been shown to be important for myogenic proliferation following muscle injury (Joe et al., 2010; Heredia et al., 2013; Biferali et al., 2019).

**FIGURE 5:**
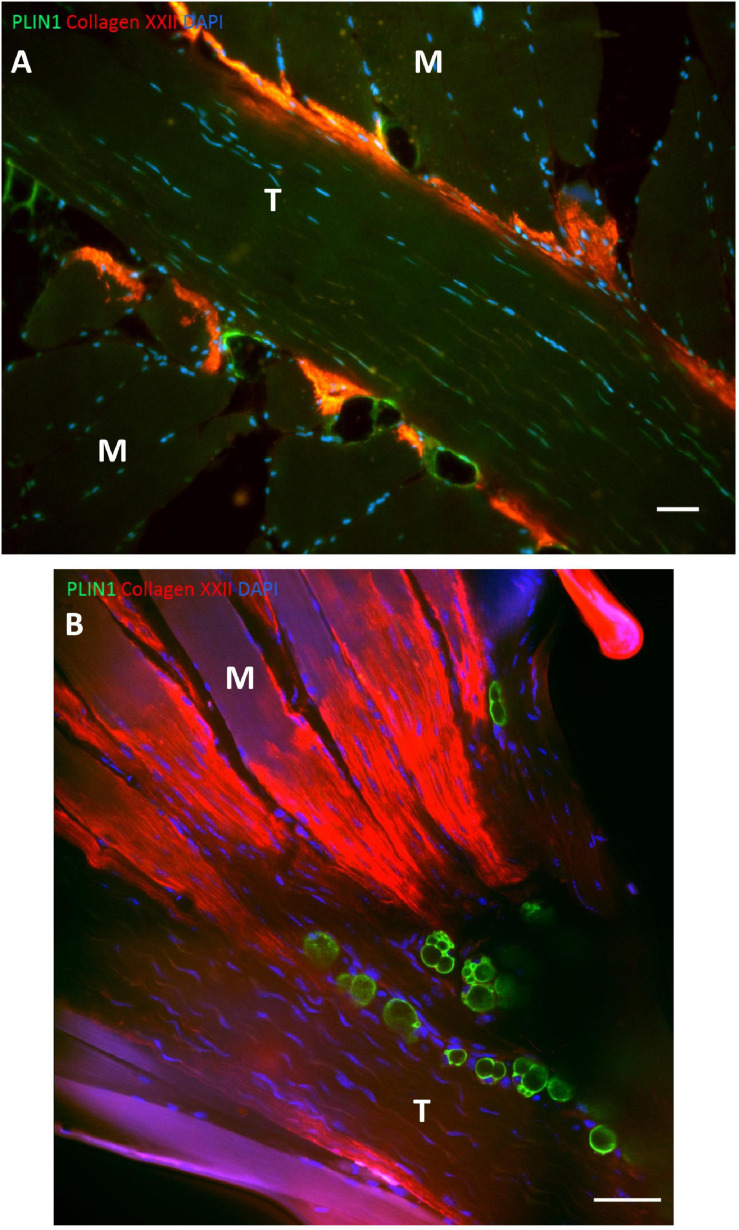
Adipocytes at human and mouse MTJ’s. **(A)** The figure shows a section from human semitendinosus muscle with the tendon (T) in the middle and the muscle fibers (M) approaching from both sides. The MTJ is identified by collagen XXII immunofluorescent staining (red) and the adipocytes are stained with PLIN1 (green), and nuclei are stained with DAPI (blue). Adipocytes are located very close to the MTJ. Scale bar is 50 μm. **(B)** Mouse soleus MTJ in confocal microscopy showing adipocytes (PLIN = green) and collagen XXII indicating the MTJ (red). Scale bar is 50 μm. Images are reprinted with permission from Jakobsen et al. (2020).

It has recently been demonstrated that adipocytes exist in high numbers in close relation to the MTJ in humans and mice (Figure 5; Jakobsen et al., 2020). Adipocytes in skeletal muscle is normally a sign of poor muscle function and insulin resistance (Vettor et al., 2009), but at the MTJ adipocytes are present in healthy humans and animals. The MTJ has previously been reported to contain a high mitochondrial content indicating an increased oxidative phosphorylation but since the adipocytes are located extracellularly, they might not be involved in the energy metabolism of the muscle fibers (Eisenberg and Milton, 1984). However, since adipocytes are known to be capable of producing various cytokines involved in numerous processes throughout the body (Ronti et al., 2006), they may play a role in the highly active remodeling process at the MTJ or in the regeneration following injury.

The regeneration and repair following muscle strain injury is a complex process which is not fully understood yet. However, following injury there is a rapid increase in inflammatory cells, macrophages and neutrophils (Butterfield et al., 2006). Macrophages initially enter the injured region as pro-inflammatory M1 macrophages, capable of phagocytosis and secretion of pro-inflammatory cytokines. As they decline in concentration, the anti-inflammatory M2 macrophages increase to aid in the repair of the injured tissue (St. Pierre and Tidball, 1994). By secreting cytokines, e.g., platelet derived growth factor (PDGF) macrophages activate fibroblasts, thereby ensuring a restoration of the ECM and formation of fibrosis (Kovacs and Dipietro, 1994). Without proper coordination between the macrophages, fibroblasts and satellite cells, optimal restoration of the tissue cannot be accomplished resulting in excessive formation of fibrosis and impaired muscle function. In skin lesions the interplay between adipocytes and fibroblasts is important for optimal healing (Schmidt and Horsley, 2013). This has not been confirmed in muscle yet, but the presence of adipocytes at the MTJ and the finding of cytokines involved in adipogenesis in the exudate of human MTJ following injury, suggests a prominent role for this cell-type in the repair of the MTJ (Bayer et al., 2019).

## Strain Injuries

Muscle injuries can be classified as either direct or indirect. The most common indirect type is the strain injury, defined as a muscular trauma affecting the tissue structure with various degree of tear (Mueller-Wohlfahrt et al., 2013). Strain injuries are among the most common injuries in a wide range of sports affecting various muscles (Orchard and Seward, 2002; Ekstrand et al., 2011; Opar et al., 2014; Ishøi et al., 2019). They are most dominantly located at the MTJ and show as small disruptions and edema between the muscle and tendon on ultrasound and MRI imaging (De Smet et al., 1990; De Smet and Best, 2000).

Muscles which are often exposed to rapid stretch during powerful movements, such as the hamstrings, rectus femoris, hip-adductor and calf muscles, are most susceptible to strain injury (Ekstrand et al., 2011; Eckard et al., 2017; Green and Pizzari, 2017; Green et al., 2020; Hultman et al., 2020). Generally, these injuries occur during activities where the muscle is either passively over-stretched or during fast eccentric contractions where the MTJ is exposed to high loads during lengthening (Askling et al., 2008; Danielsson et al., 2020; Huygaerts et al., 2020).

In the hamstring muscles two types of injury mechanisms have been identified: the stretch-type injury and sprinting-type injury. The stretch-type commonly occurs during kicking activities or when the hip is flexed while the leg is stretched, for example during pick-up of a ball in American type football (Hagel, 2005). The sprint-type injury is named after its occurrence during sprinting, and it mostly affects the proximal part of the biceps femoris long head (Ekstrand et al., 2011; Fiorentino et al., 2014; Huygaerts et al., 2020). It is suggested that the late swing phase and the early stance phase is when the hamstrings are subjected to the highest loads during running (Chumanov et al., 2011; Schache et al., 2012; Fiorentino et al., 2014). During these phases the hamstring muscles are stretched while contracting rapidly.

Similar to the hamstring strain injuries both rectus femoris and adductor longus injuries are frequently seen during kicking and sprinting activities where the muscles are exposed to rapid stretch while contracting affecting athletes in multiple disciplines where kicking and sprinting activities are involved (Mendiguchia et al., 2013; Serner et al., 2015, 2019). Strain injury to the calf muscles, “Tennis-Leg,” is seen in the medial head of gastrocnemius as well as the soleus, probably depending on injury mechanism (Delgado et al., 2002; Green et al., 2020). The mechanism of tennis leg has not been fully elucidated, but it is thought to occur during the late stance phase in sprinting activities where the knee is extended while a strong plantar flexion is performed by the gastrocnemius (Orchard et al., 2002).

Eccentric contractions can potentially cause muscle damage due to stretching of the sarcomeres, leading to fewer connections between actin and myosin and a suboptimal length-tension relationship (Gordon et al., 1966; Morgan, 1990; Dueweke et al., 2017). However, a large force production itself is not sufficient to induce strain injury in otherwise healthy MTJ (Lieber and Friden, 1993; Garrett, 1996). This indicates that in addition to a strong contraction of the sarcomeres, a forceful pull on the muscle provided by the tendon is needed to create a strain injury where the strength of the adhesion between these two tissues is exceeded. This is also the case in the previously described injury mechanisms for the most frequent strain injuries where a rapid stretch is seen together with a strong muscle contraction. Experimentally induced strain injuries in animal muscles, where the muscles are pulled while stimulated to contract, have shown that a disruption occurs either in the distal part of the muscle fibers or at the basal lamina between the muscle fiber and tendon (Tidball, 1984; Tidball and Chan, 1989), confirming that the connection between the muscle fibers and the tendon is the “weak-spot.” Unfortunately, a histologic description of a strain injury has not yet been published for humans.

## Prevention of Hamstring Strain Injuries

A recent review has summarized the current knowledge regarding diagnosis, prevention and treatment of the most common strain injuries (Ishøi et al., 2019). Therefore, the current review will focus on prevention of hamstring strain injuries, for which there is the best evidence.

Hamstring strain injuries are among the most frequent non-contact injuries in sports involving high speed running such as football, Australian rules football and athletics (Orchard and Seward, 2002; Ekstrand et al., 2011; Opar et al., 2014). It is the most common reason for inability to participate in sports. The pathogenesis of these injuries, the risk factors and possible prevention strategies have been subject for several recent studies (Mjolsnes et al., 2004; Petersen et al., 2011; Seagrave et al., 2014; Van Der Horst et al., 2015; Bourne et al., 2017). Low eccentric strength has been proposed as a risk factor for hamstring strain injury as well as short fascicle length, low hamstring to quadriceps strength ratio, hamstring tightness, muscle fatigue, poor warm-up, previous hamstring strain and age among others (Mair et al., 1996; Petersen, 2005; Opar et al., 2015; Timmins et al., 2016).

The role of muscle fatigue and fascicle length in relation to strain injury has not been fully elucidated. In a mechanistic study of strain injury, in which rabbit muscles were stretched while either being stimulated to induce contraction or left unstimulated, the stimulated muscles could produce 15% more force at failure, while the absorbed energy was 100% greater than in the unstimulated state. This suggests that muscle contractions affect the ability of the passive components to absorb force in the muscle fibers, i.e., the connective tissue (Garrett, 1996). Therefore, any condition that affects the muscle ability of the fibers to activate optimally, such as fatigue or weakness, would tend to increase the risk of injury (Mair et al., 1996). This might also explain why greater fascicle lengths are protective against strain injury since longer fascicles are capable of providing maximum force at a greater muscle length and higher velocity, meaning they are more resistant to rapid stretch (Bodine et al., 1982; Lieber and Fridén, 2000). This resistance to stretch means that fewer sarcomeres are disrupted during eccentric contractions leading to an unaffected force production and thereby an increased energy absorption from the passive structures surrounding the muscle fibers. Eccentric exercise training has been shown to increase fascicle length by adding sarcomeres in series (Lynn and Morgan, 1994), however, this has been questioned in a very recent study where there was no addition of sarcomeres in human biceps femoris muscle following 3 weeks of Nordic Hamstring exercise (Pincheira et al., 2021). Therefore, it is not clear what the mechanism behind the lengthening of the fascicles is. In addition, to induce lengthening of fascicles eccentric exercise also increases eccentric hamstring strength. Many exercise interventions have focused on eccentric exercise in the prevention of strain injuries with the Nordic Hamstring protocol being the most widely used (Askling et al., 2003; Mjolsnes et al., 2004; Arnason et al., 2008; Petersen et al., 2011; Van Der Horst et al., 2015; Bourne et al., 2018).

The original Nordic Hamstring protocol consisted of the eccentric exercise, Nordic Hamstring, periodized with increasing volume over 10 weeks (Mjolsnes et al., 2004). The Nordic Hamstring protocol reduce the incidence of hamstring strain injuries by up to 51% (Al Attar et al., 2017). The clinical effect of this type of exercise is probably due to its effects on many of the previously mentioned risk factors for strain injury. Besides increasing hamstring strength (and thereby also improving hamstring to quadriceps strength ratio) it increases the length of fascicles and probably also eccentric hamstring strength capacity (Mjolsnes et al., 2004; Bourne et al., 2017; Ishøi et al., 2018; Presland et al., 2018; Ribeiro-Alvares et al., 2018; Duhig et al., 2019; Pollard et al., 2019; Siddle et al., 2019; Whyte et al., 2019; Medeiros et al., 2020).

Future studies looking at hamstring strain injury prevention should focus on the implementation of the Nordic Hamstring protocol. Among the 32 best football clubs in Europe and 18 clubs from the Norwegian Tippeliga (where the Nordic Hamstring protocol was developed) only 10% of the clubs were compliant in 2015 with the Nordic Hamstring protocol (Bahr et al., 2015). A recent study described that athletes didn’t want to do Nordic Hamstring due to fear that they would be too sore thereby affecting their other training (Chesterton et al., 2020). This is important to address in future studies. In on our previous research we have used the Nordic Hamstring exercise on patients with anterior cruciate ligament rupture in the study of the MTJ (Jakobsen et al., 2017). Our clinical experience is that the Nordic Hamstring protocol is easily implemented, even in subjects with a low activity level, and the Delayed Onset Muscle Soreness (DOMS) rarely occur after the first couple of sessions. Therefore, the strategy to implement the Nordic Hamstring protocol in an athletic setting (Arnason et al., 2008; Petersen et al., 2011), where it is used more intensively off-season to build up strength and tolerance and less intensively in-season, seems logical.

In addition to the clinical focus on the implementation of prophylactic training, future human studies can increase our understanding of the tissue mechanisms, which besides changes in fascicle length include for instance alterations in the surface area of the MTJ and changes in the concentration of specific proteins involved in adaption of the MTJ following eccentric training. This has been explored to some extent in rats and mice, showing increases in the surface area of MTJ and concentrations of structural proteins (vinculin, talin and collagen XIV) at the MTJ following exercise (Frenette and Côté, 2000; Roffino et al., 2006; Kojima et al., 2008; Curzi et al., 2012), but it has only been sparsely studied in humans (Jakobsen et al., 2017). A deeper understanding of the effects of eccentric exercise on the MTJ at the molecular and ultrastructural level is a necessary evidence base for introducing new effective methods to prevent strain injuries.

## Perspectives

Knowledge about the human MTJ is limited. There is no standard method to obtain percutaneous biopsies from MTJ. Since the MTJ consists of two different tissues, muscle and tendon, it is necessary technically to separate samples into muscle, MTJ and tendon to investigate the metabolism in this region, and currently there is no reliable method to do this. Once these methodological challenges have been overcome, structured research in the human MTJ can be performed as basis for an understanding of strain injuries and their prevention. With the current knowledge candidate explanations of how MTJ may adapt to eccentric training are: by increasing fascicle length, increasing the surface area between muscle and tendon through enlargement of foldings, increasing concentration of structural proteins of importance for force transmission between tissues, such as dystrophin, integrins, and collagens, and increasing the lateral force transmission through the costameres. Also, in the future the interplay between macrophages, satellite cells, fibroblasts and adipocytes in the coordination of remodeling following exercise, injury and inactivity should be studied.

## What Is Already Known

The myotendinous junction (MTJ) is the interface between muscle and tendon and where force is transmitted between the two tissues. It is also a common location for strain injuries in sports. Most of these can be prevented by heavy eccentric exercise.

## What Are the New Findings

The surface area between muscle and tendon is increased by foldings of tendon into muscle, and this reduces stress between the tissues. In animals, the size and number of foldings are increased as a response to heavy training and reduced during inactivity. In humans, the muscle fibers near the MTJ show very high rates of remodeling compared to other regions of the muscle. Adipocytes are seen in high number at MTJ and might be important for the adaption of MTJ to various loading conditions.

## Author Contributions

Both authors contributed to the planning, writing, and editing of the manuscript.

## Conflict of Interest

The authors declare that the research was conducted in the absence of any commercial or financial relationships that could be construed as a potential conflict of interest.
