# Supplementary material for: The Myotendinous Junction—A Vulnerable Companion in Sports. A Narrative Review
Source: Front Physiol. 2021 Mar 26;12:635561. doi: 10.3389/fphys.2021.635561 (PMC8032995; doi:10.3389/fphys.2021.635561)
Supplement: Supplementary file 1 [file Data_Sheet_1.docx]

**The myotendinous junction – a vulnerable companion in sports. A narrative review.**

Supplementary file: Material and methods related to figure 2B

Human muscle and tendon tissue from the semitendinosus muscle was collected from surgery during reconstruction of the anterior cruciate ligament, using hamstrings tendons as graft, as reported previously(1) and prepared according to the single fiber method described by Mackey & Kjaer 2017(2). Briefly: A tendon of at least three centimeters with long muscle fibers attached was pinned in order to keep the fiber length and was immersed in Krebs-buffer with 0.1% procaine for 2 minutes followed by fixation in Zamboni fixative overnight. The fixative was then replaced by 50% glycerol in PBS and kept at +5 C overnight before storage at -20 C until further preparations.

Using a stereomicroscope the fibers with MTJ and tendon attached were carefully dissected and washed in 12-well Nunc plate filled with Immunobuffer (IB:PBS containing 50 mM glycine, 0.25% BSA, saponin, 0.05% sodium azide 0.03%). The fibers were then incubated overnight with a primary antibody (anti-collagen XXII, guinea pig anti-Collagen XXII, provided by Manuel Koch). Following washing in IB, the fibers were incubated with a secondary antibody (Alexa Fluor 568 Goat anti-guinea pig, cat. No. A-11075) and mounted on glass-slides in DAPI mounting medium following another wash.

The image in figure 2B is acquired using a Zeiss LSM710 (Carl Zeiss, Oberkochen, Germany) with the following objectives: 209/0.8 Plan-Apochromat, 409/1.3 oil DIC EC Plan-Neofluar, 639/1.4 oil DIC Plan-Apochromat.

Permissions:

The human specimens were obtained according to a protocol accepted by the local ethics committee (H-4-2011-089), and the patients signed informed consent, following the Helsinki Declaration.

References:

1. Knudsen AB, Larsen M, Mackey AL, Hjort M, Hansen KK, Qvortrup K, et al. The human myotendinous junction: an ultrastructural and 3D analysis study. Scand J Med Sci Sport. 2014/04/11. 2015;25(1):e116-23.

2. Mackey AL, Kjaer M. The breaking and making of healthy adult human skeletal muscle in vivo. Skelet Muscle. 2017 Nov 7;7(1).
